# Natural Products and Acute Myeloid Leukemia: A Review Highlighting Mechanisms of Action

**DOI:** 10.3390/nu11051010

**Published:** 2019-05-03

**Authors:** Dongwon Hwang, Minsun Kim, Hyejin Park, Myung In Jeong, Woojin Jung, Bonglee Kim

**Affiliations:** College of Korean Medicine, Kyung Hee University, 1 Hoegi-dong, Dongdaemun-gu, Seoul 130-701, Korea; d.hwang@khu.ac.kr (D.H.); red_pomme@khu.ac.kr (M.K.); hyejinpark46@khu.ac.kr (H.P.); auddls07@khu.ac.kr (M.I.J.); wj0119@khu.ac.kr (W.J.)

**Keywords:** acute myeloid leukemia, natural products, alkaloids, carotenoids, nitrogen-containing compounds, organosulfur compounds, phenolics, apoptosis

## Abstract

Recent findings have shown great potential of alternative interventions such as immunotherapy and natural products for acute myeloid leukemia (AML). This study aims to review the anti-AML effect of various natural compounds. Natural compounds were classified into five groups: alkaloids, carotenoids, nitrogen-containing compounds, organosulfur compounds or phenolics based on each compound’s chemical properties. Fifty-eight studies were collected and reviewed in this article. Phenolics are the most abundant group to have an apoptotic effect over AML cells, while other groups have also shown significant apoptotic effects. Some compounds induced apoptosis by regulating unique mechanism like human telomerase reverse transcriptase (hTERT) or laminin receptor (67LR), while others modified caspases, poly (adp-ribose) polymerase (PARP) and p53. Further study is required to identify side-effects of potent compounds and the synergistic effects of combination of two or more natural compounds or existing conventional anti-AML drugs to treat this dreadful disease.

## 1. Introduction

Acute myeloid leukemia (AML) is a cancer of the myeloid line of blood cells that infiltrate the bone marrow, blood, and other tissues [1]. Current standard intervention for AML consists of chemotherapy and stem cell transplantation. The chemotherapies include daunorubicin, idarubicin, cytarabine, and doxorubicin (showed 60% to 80% of cure rate in adults) [2]. Moreover, new drugs including vosaroxin, CPX-351, sapacitabine, SGI-110 and midostaurin are being developed for AML. Many of them are categorized into cytotoxic agents, small-molecule inhibitors, or targeted therapies [3]. Recent studies highlighted that midostaurin accompanied with standard chemotherapy significantly prolonged overall event-free survival for AML patients with a FMS-like tyrosine kinase 3 (FLT3) mutation [4]. While daunorubicin, idarubicin, cytarabine and doxorubicin remain the standard therapy for AML, the drug has been reported with a few cardiotoxic effects, a well-known risk factor for congestive heart failure [5]. AML is an incurable disease with relapse possibilities and conventional therapies have shown side-effects such as hepatotoxicity, myelosuppression, and tumor lysis syndrome [6]. Thus, we focused on discovering new potent materials for AML from natural products.

**Figure 1:**
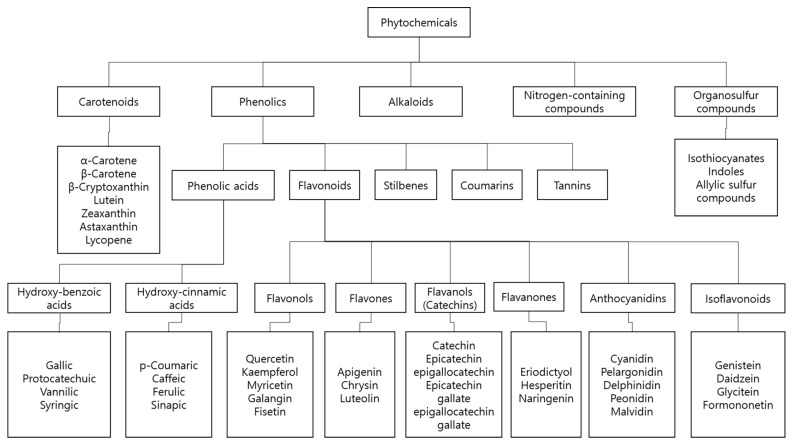
Classification of phytochemicals [7].

Natural products are extracted from abundant living organism sources. They include bioactive compounds that have the potential for prevention or treatment of major diseases and have been used as therapeutic medicine in human history [7,8,9]. Recently, they have continued to provide key scaffolds for drug development [8]. Many of these have been identified to demonstrate diverse biological actions, including anti-cancer activities [10,11]. Polyphyllin D from *Paris polyphylla* is well known for its induction of endoplasmic reticulum (ER) stress and mitochondria-mediated apoptotic pathways against lung cancer cells [12]. Resveratrol is well-known natural product that has anti-cancer effect in human cancer [13,14]. Withaferin A is a steroidal lactone from the ayurvedic plant *Withania somnifera*, which is reported to possess effective general anti-cancer activities [15]. Substantial studies highlight the anti-neoplastic effect of natural products, suggesting that they have the potential to become novel interventions for acute myeloid leukemia [16]. Furthermore, natural products or their bioactive compounds not only trigger apoptosis, but also lower the resistance against chemotherapies via in leukemic cells [17,18]. We classified natural compounds as alkaloids, carotenoids, nitrogen-containing compounds, organosulfur compounds, and phenolics in accordance with an early study (Figure 1) [7]. Carotenoids and phenolics, among these, are widely studied as chemotherapy interventions. Recent studies shed more light on the role of naturally derived products in reducing oxidative stress induced by free radicals, which is involved in a wide range of chronic diseases [19]. A well-known example of natural compound is curcumin, a yellow spice which is categorized as a phenolic compound or more specifically, a polyphenol. Curcumin is known to induce apoptosis and autophagy in prostate cancer cells [20]. In this review, potent anti-AML natural compounds were classified and reviewed by a mechanism of actions.

## 2. Methods

**Figure 2:**
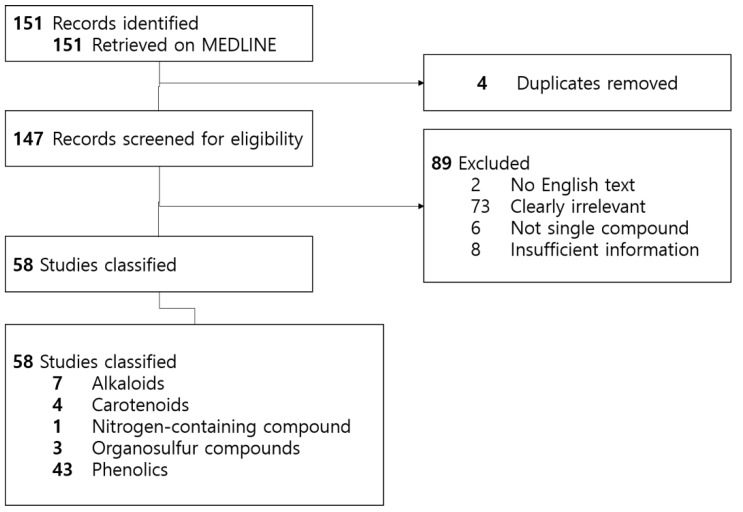
Review design flowchart.

We searched the MEDLINE database for relevant experimental literature published between September 2013 and September 2018 elucidating the apoptotic effects of natural products on specific prostate cell lines. Our search algorithm was designed based on the following criteria: each cell line including HL-60, U937, KG-1, Kasumi-1, THP-1 and their variants; related keywords such as ‘apoptosis’ and ‘natural product’ were used in the search formula. After completing the initial search, we removed duplicates, non-English literature, and studies handling multi-compound natural products such as extracts. We only took single compounds into consideration so that we could thoroughly understand how each compound exerts cytotoxicity on appropriate cells through certain signal pathways and induce apoptosis. For data unity, we have only included in vitro studies. We have classified the results into five categories, based on the phytomedicinal classification mentioned above [7]. A total of fifty-eight studies demonstrating fifty-five compounds were reviewed. We included seven alkaloids, four carotenoids, one nitrogen-containing compound, three organosulfur compounds and forty-three phenolics (Figure 2).

## 3. Natural Compounds and Acute Myeloid Leukemia

### 3.1. Alkaloids

Alkaloids contain one or more basic nitrogen atoms in a heterocyclic ring [21]. Some compounds with neutral and weakly acidic properties are also included. Many such alkaloid natural products possess cytotoxic ability towards AML cells (Table 1). Based on our findings, 4′-methoxy-5-epi-ancistecrorine A_1_, which is derived from *Ancistrocladus cochinchinensis*, expresses cytotoxicity against HL-60 cancer cells. The half maximal inhibitory concentration (IC_50_) value of the compound was around 3.8–6.2 μM against HL-60 [22]. By another alkaloid, the treatment of canthin-6-one has shown elevation of caspase-3, -8, -9 cleavage, reactive oxygen species (ROS), apoptosis signal-regulating kinase 1 (ASK1), p16, p27, p38, p53, c-Jun N-terminal kinases (JNKs), Ki-67 negative population, checkpoint kinase 2 (Chk2), H2A histone family member X (H2A.X), INK4A and Kip1, while mitochondrial membrane potential (MMP) and p- retinoblastoma protein (RB) were shown to be suppressed in Kasumi-1 cells. This result suggests that canthin-6-one effectively promotes various extrinsic and intrinsic apoptotic pathways in Kasumi-1 AML cells [23]. Curine, a natural alkaloid isolated from *Chondrodendron platyphyllum*, disrupts MMP. Additionally, curine has demonstrated potent cytotoxic effects on leukemic cell lines [24]. Indicaxanthin, isolated from cactus pear fruit, promotes cleavage of caspase-3 and poly (ADP-ribose) polymerase (PARP)-1 and free intracellular Ca^2+^ while it downregulates Ik-Bα [25]. Intermedin A extracted from *Alpinia intermedia* increases cleaved (c)-PARP and c-caspase-3 levels, thus inducing apoptosis in HL-60 cells at a dose of 30 µg/mL [26]. Increase in graft-versus-host disease (GVHD) prophylaxis has been observed in various cell lines, including KG-1, HL-60 and U937 after treatment of polyclonal anti-T-lymphocyte globulines [27]. Thalicultratine C, which originates from *Thalictrum cultratum*, downregulates MMP and induces apoptosis [28].

### 3.2. Carotenoids

Carotenoids are organic pigments that are produced by plants, algae, bacteria, and fungi. A few carotenoids have shown cytotoxic effects against AML cells (Table 2). Treatment of aromatic-tumerone separated from turmeric oil upregulates the expression of Bax, p53, c-caspase-3 and cytochrome c release [29]. Increased levels of c-caspase-3, -7, c-PARP and ROS with decrease of Bcl-xL indicate that fucoxanthin derived from *Ishige okamurae* induces apoptosis [30]. Heteronemin, which originates from *Hyrtios* sp., downregulates the activation of cytarabine-mediated factors such as mitogen-activated protein kinase (MAPK), activator protein 1 (AP-1), nuclear factor kappa-light-chain-enhancer of activated B cells (NF-κB) and c-myc [31]. Caspase-8-dependent apoptosis was observed following the treatment of isolancifolide extracted from *Actinodaphne lancifolia*. In addition, isolancifolide upregulates the levels of c-PARP, c-caspase-8, -9, while inhibiting Bid and pro-caspase-3, -8 [32].

### 3.3. Nitrogen-Containing Compounds

Not many of the nitrogen-containing compounds have been reported for their apoptotic effect against AML (Table 3). Rhizochalin (10, 20 mM for 6 or 24 h) from sponge *Rhizochalina incrustata* has shown to activate p53 and induce apoptosis in THP-1 cells [33].

### 3.4. Organosulfur Compounds

Organosulfur compounds are a subclass of natural substances that contain sulfur, widely present in the natural environment. Some of these compounds were reported for their apoptotic efficacies in AML cells (Table 4). The expression of Bax, c-caspase-3, -9, c-PARP were increased and PI3K/AKT, ERK1/2 MAPK, c-myc were suppressed after treatment of asterosaponin in HL-60 AML cells [34]. In addition, chaetocin has promoted the cleavage of caspase-3, -8 in HL-60 cells [35]. Diallyl disulfide upregulates the expression of c-myc, Sp-1, Mad1 and inhibits hTERT, decreasing telomerase activities in U937 cells [36].

### 3.5. Phenolics

Phenolics are defined as chemical compounds consisting of a hydroxyl group bonded directly to an aromatic hydrocarbon group. This is the most abundant type of phytochemicals and is thus widely studied (Table 5) [19]. Epigallocatechin-3-O-gallate (EGCG), extracted from green tea, is shown to decrease the level of 67LR, inducing apoptosis selectively in cancer cells, as compared with normal cells [37]. C-PARP, c-caspase-3, -8, -9, Bax, JNK, FasL were upregulated by vitisin B treatment, from *Vitis thunbergii* extract [38]. (R)-5-hydroxy-2-methylchroman-4-one (HMC) derived from *Cryptosporiopsis* sp. increased the expression of cyclin A, CDK1, p21, p53, PUMA, c-caspase-3, -8 -9, cytochrome c, c-PARP-1 while decreasing the expression of MMP, Bcl-2/Bax, Bid, STAT3, survivin, c-IAP-1, cyclin D1, VEGF, Bcl-xL and c-myc [39,40,41]. [8]-shogaol, originated from ginger, elevated the level of ROS, c-caspase-3, -9, c-PARP, c-DFF-45, and decreased the level of glutathione, MMP, caspase-8 and Bid [42]. Also, 5-hydroxy-6,7,3′,4′,5′-pentamethoxyflavone, extracted from *Lantana ukambensis*, induced apoptosis in U937 cells, increasing nuclear fragmentation at a dose of 10 μg/mL for 24 h, affecting nuclear apoptotic morphology [43]. Alternariol-10-methyl ether derived from *Alternaria alternate* induced various caspase cleavages, while inhibiting MMP [44]. The treatment of anthraquinone, extracted from *Hedyotis diffusa* Wild, has shown to increase the cleavage of caspase-3, -8 and -9 in HL-60 AML cells [45]. Cytochrome P450 activation was inhibited by bergenin exposure from *P. pterocarpum* [46]. Bigelovin induced both apoptosis and cell cycle arrest in a dose-dependent manner [47]. Camphene, the essential oil of *Kadsura longipedunculata*, elevated the caspase-3, -7 cleavage [48]. Activation of phosphatidylserine externalization, c-caspase-3, -8, -9, c-PARP, p38 and JNK were observed following treatment of cantharidic acid. Cantharidic acid induced apoptosis in human leukemic HL-60 cells via c-Jun N-terminal kinase-regulated caspase-3, -8, -9 activation pathway [49]. Capillin isolated from *Artemisia capillaris* Thunb. flower led to chromatin condensation and nuclear fragmentation. Additionally, the elevation of ERK1/2, JNK and cytochrome c release was observed in Capillin treated HL-60 cells [50]. Carnosic acid, separated from *Rosmarinus officinalis* polyphenol, induced apoptosis of HL-60 cells by regulating PTEN-Akt signaling pathways. Carnosic acid elevated levels of p27, c-caspase-9, PTEN, while downregulating p-BAD and p-Akt [51]. Casticin, isolated from *Vitex agnus*-castus, promoted DNA fragmentation resulting from Histone H3 phosphorylation [52]. Activation of p53, Bax, c-caspase-3, -9 and inhibition of uridine, DHODH was observed by the treatment of celastrol [53]. Mitochondrial apoptotic pathway in HL-60 and U937 cells was induced by treatment of corchorusin-D, isolated from *Corchorus acutangulus*, via elevation of DNA fragmentation and Bax expression. Corchorusin-D decreased the expression of Bcl-2 and MMP [54]. Costunolide, which originates from *Magnolia sieboldii*, induced the activation of Bcl-2, p-ERK1/2, and p-JNK, while inhibiting the generation of ROS. JNK activation is the key mechanism of constunolide-induced apoptosis [55]. Coumarin, derived from *Zanthoxylum schinifolium*, elevates Bax, c-PARP and c-caspase-3, -9 expression. Simultaneously, decrease in the levels of Bcl-2, p-ERK1/2 MAPK, p-AKT, c-myc was observed. Coumarin induces apoptosis by suppressing ERK1/2 MAPK and PIK3/AKT signaling pathways [56]. Cucurbitacin E elevates the expression level of Bax while downregulating XIAP, survivin and Mcl-1, indicating that cucurbitacin E has a role in cucurbitacin E-induced apoptosis in HL-60 cells [57]. Curcumin from turmeric upregulated p-ERK, JNK, c-caspase-3, -8 -9 and c-PARP-1 [58]. Erypoegin K, derived from *Erythrina poeppigiana*, increases the expression of c-caspase-3. Additionally, nuclear condensation and apoptotic genome DNA fragmentation were observed in erypoegin K-treated HL-60 cells [59]. Upregulation of c-PARP, c-caspase-3, -8, -9, Bid, TNFα was reported in HL-60 cells by treatment of furanodiene [60]. Gallic acid treatment led to cell death by the elevation of p53 and NF-κB, along with reduction of I-κB and GAPDH [61]. Ginsenoside Rh2 upregulated the c-caspase-3, p53 and p21 in KG1-α AML cells, demonstrating that ginsenoside Rh2 caused DNA damage in KG1-α cells via activation of intrinsic apoptotic pathway [62]. Ginsenoside Rh2 increased the expression of TGF-β, p21, p27, and reduced the expression of CDK4, CDK6, Cyclin D1, D2, D3 and E [63]. Glaucocalyxin, originated from *Rabdosia japonica* var. increased the level of c-caspase-3, -9, Bax, while decreasing the level of Bcl-2 [64]. Elevated expression level of c-caspase-3, Bax and suppressed expression of Bcl-2, c-myc, p-AKT were demonstrated following the treatment of icariside D_2_ [65]. Also, icariside II treatment promoted c-caspase-3 activity, c-PARP, PTP and SHP-1 expression in U937 cells. Suppression of Bcl-2, Bcl-x, survivin, COX-2, STAT3, JAK2 and Src was detected in icariside II treated AML cells [66]. Elevation of c-caspase-3, -9, c-PARP and inhibition of Bcl-2 were shown by treatment of isochamaejasmin [67]. Karanjin, which originates from *Fordia cauliflora*, induces cancer cell death through cell cycle arrest and induction of apoptosis on HL-60 cells [68]. Concentrations of 5-10 µg/mL of MMH01 for 24 h up-regulates the cyclin B1, Chk2, Bcl-2 and Bax, while facilitating chromatin condensation, leading to the apoptosis of U937 cells [69]. Phanginin D, the methanol extract of *Caesalpinia sappan* Linn. promoted the cleavage of caspase-3 [70]. PO-1 from *Pluchea odorata* increases the level of Cdc2, JunB, α-tubulin, NF-κB, FAK and MYPT, while inhibiting the expression of Chk2, Cdc25A, MCF-7 and CCID [71]. Proanthocyanidins (Pcys) extracted from eleven berry species elevates the cleavage of caspase-8 expression in THP-1 cells [72]. Shikonin from *Lithospermum erythrorhizon* up-regulated the generation of ROS and the cleavage of caspase-3 in HL-60 cells [73]. Down-regulation of ERP57, calreticulin, IRE-1, ATF6 and CHOP was observed in the shikonin treated AML cells. Particularly, the overexpression of ERP57 protected shikonin induced apoptosis in HL-60, whereas its knockdown lead to apoptosis activation [74]. Suppressions of FLT3 and miR-155 in THP-1 cells were observed after the exposure of silvestrol derived from *Aglaia foyeolata* [75]. Tanshinone I from *Salvia miltiorrhiza* Bunge induced the activation of Bax, c-caspase-3 and inhibition of survivin [76]. And tanshinone II A, isolated from *Salvia miltiorrhiza*, stimulated c-caspase-3 and repressed the levels of NF-κB and CCL2, promoting the apoptosis of U937 [77]. Wogonin, separated from roots of *Scutellaria baicalensis* Georgi, induced the activation of (^3^H) etoposide [78]. The treatment of wogonin increased the caspase-3 cleavage while downregulating Bcl-2, telomerase activity and c-myc [79]. Xanthatin promoted the expression level of c-caspase-3, -7 and mitigated PGE2. Xanthatin is a *Xanthium strumarium* extract, repressed the expression of Bcl-2 and HSP70 lead to β-mangostin-catalyzed apoptosis of HL-60 [80]. Conversely, the expression of the apoptotic genes such as Bax, c-caspase-3, and 9 were upregulated. These results suggest that β-mangostin showed an anti-proliferative effect on HL-60 cells and prompted the intrinsic apoptosis pathway [81].

## 4. Discussion

There are FDA approved drugs for acute myeloid leukemia (AML), which include midostaurin, liposomal cytarabine, enasidenib, gemtuzumab, and ogozamicin [82,83]. Current frontline treatment of AML is the “7 + 3” induction regimen, which includes a 7-day continuous intravenous cytarabine infusion and three daily doses of daunorubicin, especially for patients under 60 [84]. The regimen has remained as the standard cure for AML since 1973 [85,86]. Recent studies have suggested that a higher dose of cytarabine (Ara-C) and nucleoside analogue doublets may have a better effect. However, overall effect and safety of the treatment are still debatable [85,87,88]. The chemotherapies for AML can cause serious side-effects and relapse [89,90].

Targeting leukemic cells with natural products has been a big branch among various alternative attempts to treat AML. Complete cure with lower risk of relapse requires specific targeting of leukemic stem cells, and avoiding toxicity on normal hematopoietic cells. A review by Siveen et al. highlighted the various natural products that potentially target leukemic stem cells of AML. Natural products allow much lower relapse rates for AML patients [91]. Early studies have discovered many cases in which natural products exert cytotoxicity on AML, but a systematic categorization of such compounds has never been elucidated. Classifying natural products based on chemical properties can help to further understand the linkage between the chemical root of each substance and its mode of action in targeting and eliminating AML cells. Most collected natural products in this review belong to the phenolic group because phenolics are one of the most widespread substances among plants. They are involved in many different activities, including anti-Alzheimer’s disease, anti-hyperglycemia, and cancer [92,93,94]. Notwithstanding such facts, it is meaningful that many natural products, well known for their significant clinical effects, such as coumarin, curcumin, ginsenosides Rh2 and shikonin, were all phenolics. Phenolic compounds could be considered to possess advantageous structures for effectively triggering apoptotic signal pathways on specific AML cells. Some compounds in other groups have also shown great potential, although it is difficult to compare their clinical effects side by side due to different controls in experiments. Some potent compounds for treating AML include aromatic tumerone and fucoxanthin in carotenoids and asterosaponin in organosulfur compounds. While supplementary carotenoid intakes are common worldwide, further studies regarding anti-AML activities and cytotoxicity of carotenoid compounds in human are necessary [95].

One significant drawback of our study is that we have only included single compounds in the analysis. Single compounds that do not belong to any of the five categories were also excluded. Further studies with regards to the synergistic effects of conventional drugs with natural products for AML treatment are strongly required. Synergistic anti-AML effects of natural product combinations and extracts should also be studied. The interactions of phytomedicines have garnered huge interest, especially because separating single compound from extracts is difficult and natural products exert strong efficacy using low amounts of core active substance mixtures [93]. At the same time, multi-target therapies have been gaining more interest in cancer, as single-targeted therapies have shown their limitations [94,95]. Such systemic view will help researchers understand how natural products offer significant cytotoxic activities to target cells without harming adjacent environments, using low dose bioactive compounds and thereby lowering adverse effects in clinical cases [96]. Taken together, the natural products with anti-AML efficacies were categorized, and the mechanisms were reviewed in this study. These natural products have the potential to be future alternative therapies for this incurable disease, AML.

## 5. Conclusions

This study reviewed the cytotoxic effects of natural products on AML cells. We categorized the natural products and their mechanisms of actions based on a phytomedicinal classification. Natural products are promising in that they have the potential to treat AML. Further animal and clinical studies should be conducted to make use of such potential materials.

## Figures and Tables

**Table 1:** Alkaloids.

Classification	Compound	Source	Cell Line/Animal Model	Dose/Duration	Mechanism	Reference
Alkaloids	4′-methoxy-5-epi-ancistecrorine A_1_	*Ancistrocladus cochinchinensis*	HL-60	IC_50_ 3.8–6.2 μM		[22]
Alkaloids	Canthin-6-one	Various plant genera and from fungi	Kasumi-1	45 μM; 24 h	c-capase-3, -8, -9, ROS, ASK-1, p38, JNK, CHK2, p53, H2A.X, p16, INK4A, p27-Kip1 ↑	[23]
					MMP, p-RB ↓	
Alkaloids	Curine	*Chondrodendron platyphyllum*	HL-60	15 μM; 48h	MMP ↓	[24]
Alkaloids	Indicaxanthin	Cactus pear fruit	THP-1	13.5, 15, 15.5, 15.9, 16 µM (16 mM-7-KC); 12, 24 h	c-caspase-3, c-PARP-1, free intracellular Ca^2+^ ↑	[25]
					Ik-Bα ↓	
Alkaloids	Intermedin A	*Alpinia intermedia*	HL-60	23.57 ± 2.15 µg/Ml; 12 h	c-caspase-3, c-PARP ↑	[26]
Alkaloids	Polyclonal anti-T-lymphocyte globulins	Rabbit ATG- Fresenius^®^	HL-60	KG1: 100, 200, 300, 400, 500 μg/mL; 6 h	GVHD prophylaxis ↑	[27]
			KG-1	U937: 100, 200, 300, 400, 500 μg/mL; 6 h		
			U937			
Alkaloids	Thalicultratine C	*Thalictrum cultratum*	HL-60	0.06, 0.3, 1.5 μM; 24, 48, 72 h	MMP ↓	[28]

Cleaved caspase (c-caspase); reactive oxygen species (ROS); apoptosis signal-regulating kinase 1 (ASK-1); p38 mitogen-activated kinase (p38); c-Jun N-terminal kinase (JNK); checkpoint kinase 2 (CHK2); tumor suppressor p53 (p53); H2A histone family member X (H2A.X); cyclin-dependent kinase inhibitor 2A (p16); inhibitors of CDK4A (INK4A); p27-Kip1 (cyclin-dependent kinase inhibitor 1B); mitochondrial membrane potential (MMP); retinoblastoma gene product (p-RB); cleaved poly ADP-ribose polymerase 1 (c-PARP-1); nuclear factor of kappa light polypeptide gene enhancer in B-cells inhibitor, alpha (Ik-Bα); graft-versus-host disease (GVHD); ↑—up-regulation; ↓—down-regulation.

**Table 2:** Carotenoids.

Classification	Compound	Source	Cell Line/Animal Model	Dose/Duration	Mechanism	Reference
Carotenoids	Aromatic (ar)-tumerone	*Turmeric oil*	U937	40, 80, 120 µg/mL; 48 h	Bax, c-caspase-3, cytochrome c, p53 ↑	[29]
Carotenoids	Fucoxanthin	*Ishige okamurae*	HL-60	7.5, 15, 30 μg; 72 h	c-caspase-3, -7, c-PARP, ROS ↑	[30]
					Bcl-xL ↓	
Carotenoids	Heteronemin	*Hyrtios* sp.	HL-60	5 nM; 72 h	AP-1, c-myc, MAPK, NF-κB, Ras ↑	[31]
Carotenoids	Isolancifolide	*Actinodaphne lancifolia*	HL-60	HL-60: 25 μM; 2, 6, 12 h	c-caspase-8, -9, c-PARP ↑	[32]
					Bid, pro-caspase-3, -8 ↓	

Bcl-2-like protein 4 (Bax); cleaved caspase (c-caspase); tumor suppressor p53 (p53); cleaved poly ADP-ribose polymerase (c-PARP); reactive oxygen species (ROS); B-cell lymphoma-extra large (Bcl-xL); activator protein 1 (AP-1); mitogen-activated protein kinase (MAPK); nuclear factor kappa-light-chain-enhancer of activated B cells (NF-κB); Ras protein (Ras); BH3 interacting-domain death agonist (Bid); ↑—up-regulation; ↓—down-regulation.

**Table 3:** Nitrogen-containing compounds.

Classification	Compound	Source	Cell Line/Animal Model	Dose/Duration	Mechanism	Reference
Nitrogen-containing compounds	Rhizochalin	*Rhizochalina incrustata*	THP-1	10, 20 mM; 6, 24 h	p53 ↑	[33]

Tumor suppressor p53 (p53); ↑—up-regulation; ↓—down-regulation.

**Table 4:** Organosulfur compounds.

Classification	Compound	Source	Cell Line/Animal Model	Dose/Duration	Mechanism	Reference
Organosulfur compounds	Asterosaponin	*Astropecten monacanthus*	HL-60	0.01, 0.1, 1, 10, 50, 100 µM; 72 h	Bax, c-caspase-3, -9, c-PARP ↑	[34]
					AKT, Bcl-2, c-myc, ERK 1/2, MAPK, PI3K ↓	
Organosulfur compounds	Chaetocin	*Chaetomium* species fungi	HL-60	0.3 μM; 4, 24 h	c-caspase-3, -8 ↑	[35]
Organosulfur compounds	Diallyl disulfide (DADS)	*Allium sativum*	U937	25, 50, 100, 150 μM; 24 h	c-myc, Mad1, Sp-1 ↑	[36]
					hTERT ↓	

Bcl-2-like protein 4 (Bax); cleaved caspase (c-caspase); cleaved poly ADP-ribose polymerase (c-PARP); B-cell lymphoma 2 (Bcl-2); extracellular signal-regulated kinase 1, 2 (ERK1/2); mitogen-activated protein kinase (MAPK); phosphoinositide 3-kinase (PI3K); mitotic arrest deficient 1 (Mad1); Sp1 transcription factor (Sp-1); human telomerase reverse transcriptase (hTERT); ↑—up-regulation; ↓—down-regulation.

**Table 5:** Phenolics.

Classification	Compound	Source	Cell Line/Animal Model	Dose/Duration	Mechanism	Reference
Phenolics	Epigallocatechin-3-*O*-gallate (EGCG)	Green tea	HL-60	50, 100, 150, 200, 250 μg; 24 h	67LR ↓	[37]
Phenolics	Vitisin B	*Vitis thunbergii* var. taiwaniana	HL-60	HL-60: 12.5, 25 μM; 24, 36, 48 h	Bax, c-caspase-3, -8, -9, c-PARP, FasL, JNK ↑	[38]
Phenolics	(R)-5-hydroxy-2-methylchroman-4-one (HMC)	*Cryptosporiopsis* sp. H2-1, NFCCI-2856	HL-60	10, 20, 30 µg/mL; 48 h	c-caspase-3, -8, -9, c-PARP-1, CDK1, Cyclin A, cytochrome c, p21, p53, PUMA ↑	[39]
					Bax, Bcl-2, Bcl-xL, Bid, c-IAP-1, c-myc, cyclin D1, MMP, STAT3, survivin, VEGF ↓	
Phenolics	(8)-shogaol	Ginger	HL-60	30 µM	c-caspase-3, -9, c-DFF-45, c-PARP, ROS ↑	[42]
					Bid, glutathione, MMP, pro-caspase-8 ↓	
Phenolics	5-hydroxy-6,7,3′,4′,5′-pentamethoxyflavone	*Lantana ukambensis*	U937	10 μg/mL; 24 h		[43]
Phenolics	Alternariol-10-methyl ether	*Alternaria alternata*	HL-60	100, 200 µM; 72 h	c-caspases ↑	[44]
					MMP ↓	
Phenolics	Anthraquinone	*Hedyotis diffusa* Willd	HL-60	25, 50, 100, 200 μg/mL; 48 h	c-caspase-3, -8, -9 ↑	[45]
Phenolics	Bergenin	*Peltophorum pterocarpum*	HL-60	HL-60: 1–100 μM; 48 h	cytochrome P450 ↓	[46]
Phenolics	Bigelovin	*Inula helianthus-aquatia* C. Y. Wu	U937	1 µM; 24, 48 h		[47]
Phenolics	Camphene	*Kadsura longipedunculata*	HL-60	167.75 µg/mL; 24 h	c-caspase-3, -7 ↑	[48]
Phenolics	Cantharidic acid	*Blister beetles*	HL-60	10 μM; 24 h	c-caspase-3, -8, -9, c-PARP, JNK, p38 ↑	[49]
Phenolics	Capillin	*Artemisia capillaris Thunb. Flower.*	HL-60	2 μM; 6 h	cytochrome c, ERK1/2, JNK ↑	[50]
				IC_50_ 6.5 ± 2.9 µM		
					AP-1, MAPK, NF ↓	
Phenolics	Carnosic acid	*Rosmarinus officinalis* L	HL-60	HL-60: 10, 15, 20 μM/L; 24–48 h	c-caspase-9, p27, PTEN ↑	[51]
					p-AKT, p-BAD ↓	
Phenolics	Casticin	*Vitex agnus* Castus	HL-60	30, 40, 50 μg/mL; 24 h, 48 h	p-Histone H3 ↑	[52]
Phenolics	Celastrol	*Thunder God Vine*	HL-60	0.5 μM; 24 h	Bax, c-caspase -3, -9, p53 ↑	[53]
					DHODH, uridine ↓	
Phenolics	Corchorusin-D	*Corchorus acutangulus*	HL-60, U937	HL-60: 25, 50, 75, 100, 120, 150 μg/mL; 24 h	Bax ↑	[54]
				U937: 50, 75, 100, 125, 150 μg/mL; 24 h	Bcl-2, MMP ↓	
Phenolics	Costunolide	*Magnolia sieboldii*	U937	10 µM; 0.5, 1,2 h	Bcl-2, p-ERK1/2, p-JNK ↑	[55]
					Bcl-2, ROS ↓	
Phenolics	Coumarin	*Zanthoxylum schinifolium*	HL-60	HL-60: 5 μM; 24, 48 h	Bax, c-caspase -3, -9, c-PARP ↑	[56]
					Bcl-2, c-myc, p-AKT, p-ERK1/2 ↓	
Phenolics	Cucurbitacin E	*Cucurbitaceae*	HL-60	1–10 mol/L	Bax ↑	[57]
					IAP, Mcl-1, survivin ↓	
Phenolics	Curcumin	turmeric	THP-1	50 μM; 3, 6, 12 h	c-caspase-3, -8, -9, c-PARP-1, JNK, p-ERK ↑	[58]
Phenolics	Erypoegin K	*Erythrina poeppigiana*	HL-60	0.175 ± 0.004 μM; 48h	c-caspase-3 ↑	[59]
				10 μM; 24h		
Phenolics	Furanodiene	*Curcuma wenyujin*	HL-60	10, 30, 50, 70 μM; 6 h	Bid, c-caspase-3, -8, -9, c-PARP, TNFα ↑	[60]
Phenolics	Gallic acid	*Rhus chinensis*	U937	5.8, 58, 580 µM; 24 h	p53, NF-κB, ↑	[61]
					GAPDH, I-κB ↓	
Phenolics	Ginsenoside Rh2		KG-1	100, 200, 300, 400, 500 μg/mL; 6 h	c-caspase-3, p21, p53 ↑	[62]
Phenolics	Ginsenoside Rh2	*Panax ginseng*	HL-60, U937	HL-60: 10, 20, 30 μM; 24, 48, 72 h	p21, p27 ↑	[63]
				U937: 10, 20, 30 μM; 24, 48, 72 h	CDK4, CDK6, Cyclin D1, Cyclin D2, Cyclin D3, Cyclin E ↓	
Phenolics	Glaucocalyxin A	*Rabdosia japonica* var. glaucocalyx	HL-60	10.0 μg/mL; 24 h	Bax, c-caspase-3, -9 ↑	[64]
					Bcl-2 ↓	
Phenolics	Icariside D_2_	*Annona glabra* Linn	HL-60	IC_50_ 9.0 ± 1.0 µM; 72h	Bax, c-caspase -3 ↑	[65]
				9.0 µM; 24, 48 h	Bcl-2, c-myc, c-PARP, p-AKT ↓	
Phenolics	Icariside II	*Epimedium koreanum*	U937	25, 50 μM; 24, 48, 72 h	c-caspase-3, c-PARP, PTP, SHP-1 ↑	[66]
					Bcl-2, Bcl-X, COX-2, JAK2, Src, STAT3, survivin ↓	
Phenolics	Isochamaejasmin	*Stellera chamaejasme* L. (Thymelaeaceae)	HL-60	HL-60: IC_50_ 50.40 ± 1.21 μmol·L^−1^ 25 and 50 μmol·L^−1^; 48 h	c-caspase-3, -9, c-PARP ↑	[67]
					Bcl-2 ↓	
				K562: IC_50_ 24.51 ± 1.62 μmol·L^−1^		
Phenolics	Karanjin	*Fordia cauliflora*	HL-60	2, 4, 6 µM; 72 h		[68]
Phenolics	MMH01	*Antrodia cinnamomea*	U937	5, 10 µg/mL; 24 h	Bax, Bcl-2, Chk2, Cyclin B1 ↑	[69]
Phenolics	Phanginin D	*Caesalpinia sappan* Linn. (Leguminosae)	HL-60	10, 30 µM; 24, 48 h	c-caspase-3 ↑	[70]
Phenolics	PO-1	*Pluchea odorata*	HL-60	IC_50_ 8.9 μM; 72 h	α-tubulin, Cdc2, FAK, JunB, MYPT, NF-κB ↑	[71]
				25, 50, 100 μM; 24, 48, 72 h	CCID, Cdc25A, Chk2, MCF-7 ↓	
Phenolics	Proanthocyanidins	11 berry species	THP-1	50 μg/mL; 24 h	c-caspase 8 ↑	[72]
Phenolics	Shikonin	*Lithospermum erythrorhizon*	HL-60	1, 2, 5 μM; 24, 48 h	ROS, c-caspase-3 ↑	[73]
Phenolics	Shikonin	*Lithospermum erythrorhizon*	HL-60	2.5 µM; 24 h	ATF6, calreticulin, CHOP, ERp57, IRE-1 ↓	[74]
Phenolics	Silvestrol	*Aglaia foveolata*	THP-1	50 nm; 24 h	FLT3, miR-155 ↓	[75]
Phenolics	Tanshinone I	*Salvia miltiorrhiza* Bunge	HL-60	31 ± 7.1 μmol/L; 24 h,	Bax ↑	[76]
				22 ± 7.6 μmol/L; 48 h,		
					c-caspase-3, survivin ↓	
				15 ± 4.3 μmol/L; 72 h		
Phenolics	Tanshinone IIA	*Salvia miltiorrhiza*	U937	2, 3, 5, 10 µg/mL; 12 h, 24, 36, 48 h	c-caspase 3, PXR ↑	[77]
					CCL2, NF-κB ↓	
Phenolics	Wogonin	*Scutellaria baicalensis* Georgi	HL-60	10 μM; 24h	[^3^H]etoposide ↑	[78]
					P-gp ↓	
Phenolics	Wogonin	*Scutellaria baicalensis*	HL-60	10 mg/mL; 20, 25, 30, 35, 40 min	c-caspase-3 ↑	[79]
					Bcl-2, c-myc, telomerase ↓	
Phenolics	Xanthatin	*Xanthium strumarium*	HL-60	HL-60: 100 μg/mL; 48 h	c-caspase-3, -7 ↑	[80]
					PGE2 ↓	
Phenolics	β-mangostin	*Cratoxylum arborescens*	HL-60	58 µM; 24 h	Bax, c-caspase -3, -9, cytochrome c, p53 ↑	[81]
					Bcl-2, HSP70 ↓	

67 kDa laminin receptor (67LR); bcl-2-like protein 4 (Bax); cleaved caspase (c-caspase); cleaved poly ADP-ribose polymerase (c-PARP); first apoptosis signal ligand (FasL); c-Jun N-terminal kinase (JNK); cyclin dependent kinase 1 (CDK1); cyclin-dependent kinase inhibitor 1 (p21); tumor suppressor p53 (p53); p53 upregulated modulator of apoptosis (PUMA); B-cell lymphoma 2 (Bcl-2); B-cell lymphoma-extra large (Bcl-xL); BH3 interacting-domain death agonist (Bid); cellular inhibitor of apoptosis protein 1 (c-IAP-1); mitochondrial membrane potential (MMP); signal transducer and activator of transcription 3 (STAT3); vascular endothelial growth factor (VEGF); cleaved DNA fragmentation factor 45 (c-DFF-45); reactive oxygen species (ROS); p38 mitogen activated protein (p38); extracellular signal-regulated kinase 1, 2 (ERK1/2); activator protein 1 (AP-1); mitogen-activated protein kinase (MAPK); nuclear factor (NF); cyclin-dependent kinase inhibitor 1B (p27); phosphatase and tensin homolog (PTEN); phospho-protein kinase B (p-AKT); phospho-Bcl-2 associated death (p-BAD); phospho-histone H3 (p-Histone H3); dihydroorotate dehydrogenase (DHODE); phospho-extracellular signal-regulated kinase 1, 2 (p-ERK1/2); phospho-c-Jun N-terminal kinase (p-JNK); inhibitor of apoptosis protein (IAP); myeloid cell leukemia 1 (Mcl-1); tumor necrosis factor alpha (TNFα); nuclear factor kappa-light-chain-enhancer of activated B cells (NF-κB); glyceraldehyde 3-phosphate dehydrogenase (GAPDH); cyclin dependent kinase (CDK); protein tyrosine phosphatase (PTP); Src homology 2 domain-containing protein tyrosine phosphatase 1 (SHP-1); B-cell lymphoma X (Bcl-X); cyclooxygenase 2 (COX-2); Janus kinase 2 (JAK2); Src family of the tyrosine kinases (Src); signal transducer and activator of transcription 3 (STAT3); checkpoint kinase 2 (Chk2); cell division control 2 (Cdc2), focal adhesion kinase (FAK), JunB proto-oncogene (JunB); myosin phosphatase targeting protein (MYPT); cell culture infectious dose (CCID); M-phase inducer phosphatase 1 (Cdc25A); activating transcription factor 6 (ATF6); CCAAT-enhancer-binding protein homologous protein (CHOP); protein disulfide-isomerase A3 (ERp57); inositol-requiring enzyme 1 (IRE-1); fms like tyrosine kinase 3 (FLT3); micro RNA 155 (miR-155); pregnane X receptor (PXR); C-C Motif Chemokine ligand 2 (CCL2); P-glycoprotein (P-gp); prostaglandin E2 (PGE2); heat shock protein 70 (HSP70); ↑—up-regulation; ↓—down-regulation.

## References

[1] Dohner H., Weisdorf D.J., Bloomfield C.D. (2015). Acute myeloid leukemia. N. Engl. J. Med..

[2] Dombret H., Gardin C. (2016). An update of current treatments for adult acute myeloid leukemia. Blood.

[3] Kadia T.M., Ravandi F., Cortes J., Kantarjian H. (2016). New drugs in acute myeloid leukemia. Ann. Oncol..

[4] Stone R.M., Mandrekar S.J., Sanford B.L., Laumann K., Geyer S., Bloomfield C.D., Thiede C., Prior T.W., Döhner K., Marcucci G. (2017). Midostaurin plus Chemotherapy for Acute Myeloid Leukemia with a FLT3 Mutation. N. Engl. J. Med..

[5] Jarfelt M., Andersen N.H., Hasle H. (2016). Is it possible to cure childhood acute myeloid leukaemia without significant cardiotoxicity?. Br. J. Haematol..

[6] Watts J., Nimer S. (2018). Recent advances in the understanding and treatment of acute myeloid leukemia. F1000Research.

[7] Liu R.H. (2004). Potential synergy of phytochemicals in cancer prevention: Mechanism of action. J. Nutr..

[8] Katz L., Baltz R.H. (2016). Natural product discovery: Past, present, and future. J. Ind. Microbiol. Biotechnol..

[9] Kumar A., Premoli M., Aria F., Bonini S.A., Maccarinelli G., Gianoncelli A., Memo M., Mastinu A. (2019). Cannabimimetic plants: Are they new cannabinoidergic modulators?. Planta.

[10] Drahl C., Cravatt B.F., Sorensen E.J. (2005). Protein-reactive natural products. Angew. Chem. Int. Ed. Engl..

[11] Bonini S.A., Premoli M., Tambaro S., Kumar A., Maccarinelli G., Memo M., Mastinu A. (2018). Cannabis sativa: A comprehensive ethnopharmacological review of a medicinal plant with a long history. J. Ethnopharmacol..

[12] Siu F.-M., Ma D.-L., Cheung Y.-W., Lok C.-N., Yan K., Yang Z., Yang M., Xu S., Ko B.C.-B., He Q.-Y. (2008). Proteomic and transcriptomic study on the action of a cytotoxic saponin (Polyphyllin D): Induction of endoplasmic reticulum stress and mitochondria-mediated apoptotic pathways. Proteomics.

[13] Park J.-W., Woo K.J., Lee J.-T., Lim J.H., Lee T.-J., Kim S.H., Choi Y.H., Kwon T.K. (2007). Resveratrol induces pro-apoptotic endoplasmic reticulum stress in human colon cancer cells. Oncol. Rep..

[14] Carter L.G., D’Orazio J.A., Pearson K.J. (2014). Resveratrol and cancer: Focus on in vivo evidence. Endocr. Relat. Cancer.

[15] Grossman E.A., Ward C.C., Spradlin J.N., Bateman L.A., Huffman T.R., Miyamoto D.K., Kleinman J.I., Nomura D.K. (2017). Covalent Ligand Discovery Against Druggable Hotspots Targeted by Anti-Cancer Natural Products. Cell Chem. Biol..

[16] Roomi M.W., Kalinovsky T., Roomi N.W., Niedzwiecki A., Rath M. (2015). In vitro and in vivo inhibition of human Fanconi anemia head and neck squamous carcinoma by a phytonutrient combination. Int. J. Oncol..

[17] Kim C., Kim B. (2018). Anti-Cancer Natural Products and Their Bioactive Compounds Inducing ER Stress-Mediated Apoptosis: A Review. Nutrients.

[18] Premoli M., Aria F., Bonini S.A., Maccarinelli G., Gianoncelli A., Della Pina S., Tambaro S., Memo M., Mastinu A. (2019). Cannabidiol: Recent advances and new insights for neuropsychiatric disorders treatment. Life Sci..

[19] Ames B.N., Gold L.S. (1991). Endogenous mutagens and the causes of aging and cancer. Mutat. Res..

[20] Yang C., Ma X., Wang Z., Zeng X., Hu Z., Ye Z., Shen G. (2017). Curcumin induces apoptosis and protective autophagy in castration-resistant prostate cancer cells through iron chelation. Drug Des. Dev. Ther..

[21] Barbieri R., Coppo E., Marchese A., Daglia M., Sobarzo-Sánchez E., Nabavi S.F., Nabavi S.M. (2017). Phytochemicals for human disease: An update on plant-derived compounds antibacterial activity. Microbiol. Res..

[22] Lien le Q., Linh T.M., Giang V.H., Mai N.C., Nhiem N.X., Tai B.H., Cuc N.T., Anh Hle T., Ban N.K., Minh C.V. (2016). New naphthalene derivatives and isoquinoline alkaloids from ancistrocladus cochinchinensis with their anti-proliferative activity on human cancer cells. Bioorg. Med. Chem. Lett..

[23] Vieira Torquato H.F., Ribeiro-Filho A.C., Buri M.V., Araujo Junior R.T., Pimenta R., de Oliveira J.S., Filho V.C., Macho A., Paredes-Gamero E.J., de Oliveira Martins D.T. (2017). Canthin-6-one induces cell death, cell cycle arrest and differentiation in human myeloid leukemia cells. Biochim. Biophys. Acta.

[24] Dantas B.B., Faheina-Martins G.V., Coulidiati T.H., Bomfim C.C.B., Dias C.D.S., Barbosa-Filho J.M., Araújo D.A.M. (2015). Effects of curine in HL-60 leukemic cells: Cell cycle arrest and apoptosis induction. J. Nat. Med..

[25] Tesoriere L., Attanzio A., Allegra M., Gentile C., Livrea M.A. (2013). Phytochemical indicaxanthin suppresses 7-ketocholesterol-induced thp-1 cell apoptosis by preventing cytosolic ca(2+) increase and oxidative stress. Br. J. Nutr..

[26] Chen L.G., Su P.J., Tsai P.W., Yang L.L., Wang C.C. (2017). Intermedin a, a new labdane diterpene isolated from alpinia intermedia, prolonged the survival time of p-388d1 tumor-bearing cdf1 mice. Planta Med..

[27] Grüllich C., Ziegler C., Finke J. (2009). Rabbit Anti T-Lymphocyte Globulin Induces Apoptosis in Peripheral Blood Mononuclear Cell Compartments and Leukemia Cells, While Hematopoetic Stem Cells Are Apoptosis Resistant. Biol. Blood Marrow Transplant..

[28] Li D.-H., Li J.-Y., Xue C.-M., Han T., Sai C.-M., Wang K.-B., Lu J.-C., Jing Y.-K., Hua H.-M., Li Z.-L. (2017). Antiproliferative Dimeric Aporphinoid Alkaloids from the Roots of Thalictrum cultratum. J. Nat. Prod..

[29] Lee Y.-K. (2009). Activation of apoptotic protein in U937 cells by a component of turmeric oil. BMB Rep..

[30] Kim K.N., Heo S.J., Kang S.M., Ahn G., Jeon Y.J. (2010). Fucoxanthin induces apoptosis in human leukemia hl-60 cells through a ros-mediated bcl-xl pathway. Toxicol. In Vitro.

[31] Saikia M., Retnakumari A.P., Anwar S., Anto N.P., Mittal R., Shah S., Pillai K.S., Balachandran V.S., Peter V., Thomas R. (2018). Heteronemin, a marine natural product, sensitizes acute myeloid leukemia cells towards cytarabine chemotherapy by regulating farnesylation of Ras. Oncotarget.

[32] Choi J.J., Kwon O.-K., Oh S.-R., Lee H.-K., Ahn K.-S. (2012). The effect of isolancifolide on the apoptosis in HL-60 cells through caspase-8-dependent and -independent pathways. Arch. Pharmacal Res..

[33] Fedorov S.N., Makarieva T.N., Guzii A.G., Shubina L.K., Kwak J.Y., Stonik V.A. (2009). Marine two-headed sphingolipid-like compound rhizochalin inhibits egf-induced transformation of jb6 p+ cl41 cells. Lipids.

[34] Thao N.P., Luyen B.T.T., Kim E.-J., Kang H.-K., Kim S., Cuong N.X., Nam N.H., Van Kiem P., Van Minh C., Kim Y.H. (2014). Asterosaponins from the Starfish Astropecten monacanthus Suppress Growth and Induce Apoptosis in HL-60, PC-3, and SNU-C5 Human Cancer Cell Lines. Boil. Pharm. Bull..

[35] Teng Y., Iuchi K., Iwasa E., Fujishiro S., Hamashima Y., Dodo K., Sodeoka M. (2010). Unnatural enantiomer of chaetocin shows strong apoptosis-inducing activity through caspase-8/caspase-3 activation. Bioorgan. Med. Chem. Lett..

[36] Dasgupta P., Sengupta S.B. (2015). Role of diallyl disulfide-mediated cleavage of c-Myc and Sp-1 in the regulation of telomerase activity in human lymphoma cell line U937. Nutrition.

[37] Okada N., Tanabe H., Tazoe H., Ishigami Y., Fukutomi R., Yasui K., Isemura M. (2009). Differentiation-associated alteration in sensitivity to apoptosis induced by (−)-epigallocatechin-3-*O*-gallate in HL-60 cells. Biomed. Res..

[38] Wu S.S., Chen L.G., Lin R.J., Lin S.Y., Lo Y.E., Liang Y.C. (2013). Cytotoxicity of (−)-vitisin b in human leukemia cells. Drug Chem. Toxicol..

[39] Pathania A.S., Guru S.K., Ashraf N.U., Riyaz-Ul-Hassan S., Ali A., Tasduq S.A., Malik F., Bhushan S. (2015). A novel stereo bioactive metabolite isolated from an endophytic fungus induces caspase dependent apoptosis and STAT-3 inhibition in human leukemia cells. Eur. J. Pharmacol..

[40] McMahon C.M., Luger S.M. (2019). Maintenance therapy in acute myeloid leukemia: What is the future?. Semin. Hematol..

[41] Christman L.M., Dean L.L., Allen J.C., Godinez S.F., Toomer O.T. (2019). Peanut skin phenolic extract attenuates hyperglycemic responses in vivo and in vitro. PLoS ONE.

[42] Shieh P.-C., Chen Y.-O., Kuo D.-H., Chen F.-A., Tsai M.-L., Chang I.-S., Wu H., Sang S., Ho C.-T., Pan M.-H. (2010). Induction of Apoptosis by [8]-shogaol via Reactive Oxygen Species Generation, Glutathione Depletion and Caspase Activation in Human Leukemia Cells. J. Agric. Food Chem..

[43] Sawadogo W.R., Cerella C., Al-Mourabit A., Moriou C., Teiten M.-H., Guissou I.P., Dicato M., Diederich M. (2015). Cytotoxic, Antiproliferative and Pro-Apoptotic Effects of 5-Hydroxyl-6,7,3′,4′,5′-Pentamethoxyflavone Isolated from Lantana ukambensis. Nutrients.

[44] Devari S., Jaglan S., Kumar M., Deshidi R., Guru S., Bhushan S., Kushwaha M., Gupta A.P., Gandhi S.G., Sharma J.P. (2014). Capsaicin production by Alternaria alternata, an endophytic fungus from Capsicum annum; LC–ESI–MS/MS analysis. Phytochemistry.

[45] Kuo Y.J., Yang J.S., Lu C.C., Chiang S.Y., Lin J.G., Chung J.G. (2015). Ethanol extract of hedyotis diffusa willd upregulates g0/g1 phase arrest and induces apoptosis in human leukemia cells by modulating caspase cascade signaling and altering associated genes expression was assayed by cdna microarray. Environ. Toxicol..

[46] Zhang J., Nishimoto Y., Tokuda H., Suzuki N., Yasukawa K., Kitdamrongtham W., Akazawa H., Manosroi A., Manosroi J., Akihisa T. (2013). Cancer Chemopreventive Effect of Bergenin from *Peltophorum pterocarpum* Wood. Chem. Biodivers..

[47] Zeng G.Z., Tan N.H., Ji C.J., Fan J.T., Huang H.Q., Han H.J., Zhou G.B. (2009). Apoptosis inducement of bigelovin from inula helianthus-aquatica on human leukemia u937 cells. Phytother. Res..

[48] Mulyaningsih S., Youns M., El-Readi M.Z., Ashour M.L., Nibret E., Sporer F., Herrmann F., Reichling J., Wink M., El-Readi M.Z. (2010). Biological activity of the essential oil of Kadsura longipedunculata (Schisandraceae) and its major components. J. Pharm. Pharmacol..

[49] Wang S.-C., Chow J.-M., Chien M.-H., Lin C.-W., Chen H.-Y., Hsiao P.-C., Yang S.-F. (2018). Cantharidic acid induces apoptosis of human leukemic HL-60 cells via c-Jun N-terminal kinase-regulated caspase-8/-9/-3 activation pathway. Environ. Toxicol..

[50] Masuda Y., Asada K., Satoh R., Takada K., Kitajima J. (2015). Capillin, a major constituent of Artemisia capillaris Thunb. flower essential oil, induces apoptosis through the mitochondrial pathway in human leukemia HL-60 cells. Phytomedicine.

[51] Wang R., Cong W.-H., Guo G., Li X.-X., Chen X.-L., Yu X.-N., Li H. (2012). Synergism between carnosic acid and arsenic trioxide on induction of acute myeloid leukemia cell apoptosis is associated with modulation of PTEN/Akt signaling pathway. Chin. J. Integr. Med..

[52] Kikuchi H., Yuan B., Yuhara E., Imai M., Furutani R., Fukushima S., Hazama S., Hirobe C., Ohyama K., Takagi N. (2014). Involvement of histone H3 phosphorylation via the activation of p38 MAPK pathway and intracellular redox status in cytotoxicity of HL-60 cells induced by Vitex agnus-castus fruit extract. Int. J. Oncol..

[53] Zhang X., Yang J., Chen M., Li L., Huan F., Li A., Liu Y., Xia Y., Duan J.-A., Ma S. (2016). Metabolomics profiles delineate uridine deficiency contributes to mitochondria-mediated apoptosis induced by celastrol in human acute promyelocytic leukemia cells. Oncotarget.

[54] Mallick S., Ghosh P., Samanta S.K., Kinra S., Pal B.C., Gomes A., Vedasiromoni J.R. (2010). Corchorusin-d, a saikosaponin-like compound isolated from corchorus acutangulus lam., targets mitochondrial apoptotic pathways in leukemic cell lines (hl-60 and u937). Cancer Chemother. Pharmacol..

[55] Choi J.-H., Lee K.-T. (2009). Costunolide-Induced Apoptosis in Human Leukemia Cells: Involvement of c-Jun N-Terminal Kinase Activation. Biol. Pharm. Bull..

[56] Xie L., Kang H., Xu Q., Chen M.J., Liao Y., Thiyagarajan M., O’Donnell J., Christensen D.J., Nicholson C., Iliff J.J. (2013). Sleep Drives Metabolite Clearance from the Adult Brain. Science.

[57] Li Y., Wang R., Ma E., Deng Y., Wang X., Xiao J., Jing Y. (2010). The induction of g2/m cell-cycle arrest and apoptosis by cucurbitacin e is associated with increased phosphorylation of eif2alpha in leukemia cells. Anti-Cancer Drugs.

[58] Yang C.-W., Chang C.-L., Lee H.-C., Chi C.-W., Pan J.-P., Yang W.-C. (2012). Curcumin induces the apoptosis of human monocytic leukemia THP-1 cells via the activation of JNK/ERK Pathways. BMC Complement. Altern. Med..

[59] Hikita K., Hattori N., Takeda A., Yamakage Y., Shibata R., Yamada S., Kato K., Murata T., Tanaka H., Kaneda N. (2018). Potent apoptosis-inducing activity of erypoegin k, an isoflavone isolated from erythrina poeppigiana, against human leukemia hl-60 cells. J. Nat. Med..

[60] Ma E., Wang X., Li Y., Sun X., Tai W., Li T., Guo T. (2008). Induction of apoptosis by furanodiene in HL60 leukemia cells through activation of TNFR1 signaling pathway. Cancer Lett..

[61] Kim N.-S., Jeong S.-I., Hwang B.-S., Lee Y.-E., Kang S.-H., Lee H.-C., Oh C.-H. (2011). Gallic Acid Inhibits Cell Viability and Induces Apoptosis in Human Monocytic Cell Line U937. J. Med. Food.

[62] You Z., Chen D., Wei Q., Zhao L., Xia J., Li D., Li J. (2014). Ginsenoside Rh2 inhibits proliferation and promotes apoptosis of leukemia KG1-α cells. Xi Bao Yu Fen Zi Mian Yi Xue Za Zhi = Chin. J. Cell. Mol. Immunol..

[63] Chung K.S., Cho S.H., Shin J.S., Kim D.H., Choi J.H., Choi S.Y., Rhee Y.K., Hong H.D., Lee K.T. (2013). Ginsenoside rh2 induces cell cycle arrest and differentiation in human leukemia cells by upregulating tgf-beta expression. Carcinogenesis.

[64] Saxena A., Saxena A.K., Singh J., Bhushan S. (2010). Natural antioxidants synergistically enhance the anticancer potential of AP9-cd, a novel lignan composition from *Cedrus deodara* in human leukemia HL-60 cells. Chem.-Biol. Interact..

[65] Hien N.T., Nhiem N.X., Yen D.T., Hang D.T., Tai B.H., Quang T.H., Tuan Anh H.L., Kiem P.V., Minh C.V., Kim E.J. (2015). Chemical constituents of the annona glabra fruit and their cytotoxic activity. Pharm. Biol..

[66] Kang S.-H., Jeong S.-J., Kim S.-H., Kim J.-H., Jung J.H., Koh W., Kim J.H., Kim D.K., Chen C.-Y., Kim S.-H. (2012). Icariside II Induces Apoptosis in U937 Acute Myeloid Leukemia Cells: Role of Inactivation of STAT3-Related Signaling. PLoS ONE.

[67] Zhang S.-D., Shan L., Li W., Li H.-L., Zhang W.-D. (2015). Isochamaejasmin induces apoptosis in leukemia cells through inhibiting Bcl-2 family proteins. Chin. J. Nat. Med..

[68] Guo J.-R., Chen Q.-Q., Lam C.W.-K., Zhang W. (2015). Effects of karanjin on cell cycle arrest and apoptosis in human A549, HepG2 and HL-60 cancer cells. Biol. Res..

[69] Chen Y.J., Chou C.J., Chang T.T. (2009). Compound mmh01 possesses toxicity against human leukemia and pancreatic cancer cells. Toxicol. In Vitro.

[70] Tran M.H., Nguyen M.T., Nguyen H.D., Nguyen T.D., Phuong T.T. (2015). Cytotoxic constituents from the seeds of Vietnamese *Caesalpinia sappan*. Pharm. Biol..

[71] Blaschke M., McKinnon R., Nguyen C.H., Holzner S., Zehl M., Atanasov A.G., Schelch K., Krieger S., Diaz R., Frisch R. (2015). A eudesmane-type sesquiterpene isolated from *Pluchea odorata* (L.) Cass. combats three hallmarks of cancer cells: Unrestricted proliferation, escape from apoptosis and early metastatic outgrowth in vitro. Mutat. Res..

[72] Minker C., Duban L., Karas D., Järvinen P., Lobstein A., Muller C.D. (2015). Impact of Procyanidins from Different Berries on Caspase 8 Activation in Colon Cancer. Oxidative Med. Cell. Longev..

[73] Duan D., Zhang B., Yao J., Liu Y., Fang J. (2014). Shikonin targets cytosolic thioredoxin reductase to induce ROS-mediated apoptosis in human promyelocytic leukemia HL-60 cells. Free Radic. Biol. Med..

[74] Trivedi R., Müller G.A., Rathore M.S., Mishra D.P., Dihazi H. (2016). Anti-Leukemic Activity of Shikonin: Role of ERP57 in Shikonin Induced Apoptosis in Acute Myeloid Leukemia. Cell. Physiol. Biochem..

[75] Alachkar H., Santhanam R., Harb J.G., Lucas D.M., Oaks J.J., Hickey C.J., Pan L., Kinghorn A.D., Caligiuri M.A., Perrotti D. (2013). Silvestrol exhibits significant in vivo and in vitro antileukemic activities and inhibits FLT3 and miR-155 expressions in acute myeloid leukemia. J. Hematol. Oncol..

[76] Liu J.-J., Liu W.-D., Yang H.-Z., Zhang Y., Fang Z.-G., Liu P.-Q., Lin D.-J., Xiao R.-Z., Hu Y., Wang C.-Z. (2010). Inactivation of PI3k/Akt signaling pathway and activation of caspase-3 are involved in tanshinone I-induced apoptosis in myeloid leukemia cells in vitro. Ann. Hematol..

[77] Liu C., Li J., Wang L., Wu F., Huang L., Xu Y., Ye J., Xiao B., Meng F., Chen S. (2012). Analysis of tanshinone IIA induced cellular apoptosis in leukemia cells by genome-wide expression profiling. BMC Complement. Altern. Med..

[78] Enomoto R., Koshiba C., Suzuki C., Lee E. (2011). Wogonin potentiates the antitumor action of etoposide and ameliorates its adverse effects. Cancer Chemother. Pharmacol..

[79] Huang S.-T., Wang C.-Y., Yang R.-C., Chu C.-J., Wu H.-T., Pang J.-H.S. (2010). Wogonin, an active compound in Scutellaria baicalensis, induces apoptosis and reduces telomerase activity in the HL-60 leukemia cells. Phytomedicine.

[80] Nibret E., Youns M., Krauth-Siegel R.L., Wink M. (2011). Biological Activities of Xanthatin from Xanthium strumarium Leaves. Phytother. Res..

[81] Omer F.A.A., Hashim N.B.M., Ibrahim M.Y., Dehghan F., Yahayu M., Karimian H., Salim L.Z.A., Mohan S. (2017). Beta-mangostin from Cratoxylum arborescens activates the intrinsic apoptosis pathway through reactive oxygen species with downregulation of the HSP70 gene in the HL60 cells associated with a G_0_/G_1_ cell-cycle arrest. Tumor Biol..

[82] Baron J., Wang E.S. (2018). Gemtuzumab ozogamicin for the treatment of acute myeloid leukemia. Expert Rev. Clin. Pharmacol..

[83] Click Z.R., Seddon A.N., Bae Y.R., Fisher J.D., Ogunniyi A. (2018). New Food and Drug Administration-Approved and Emerging Novel Treatment Options for Acute Myeloid Leukemia. Pharmacotherapy.

[84] Yates J.W., Wallace H.J., Ellison R.R., Holland J.F. (1973). Cytosine arabinoside (NSC-63878) and daunorubicin (NSC-83142) therapy in acute nonlymphocytic leukemia. Cancer Chemother. Rep..

[85] Kadia T.M., Ravandi F., O’Brien S., Cortes J., Kantarjian H.M. (2015). Progress in acute myeloid leukemia. Clin. Lymphoma Myeloma Leuk..

[86] Dohner H., Estey E.H., Amadori S., Appelbaum F.R., Buchner T., Burnett A.K., Dombret H., Fenaux P., Grimwade D., Larson R.A. (2010). Diagnosis and management of acute myeloid leukemia in adults: Recommendations from an international expert panel, on behalf of the European leukemianet. Blood.

[87] Burnett A.K., Russell N.H., Hills R.K., Hunter A.E., Kjeldsen L., Yin J., Gibson B.E., Wheatley K., Milligan D. (2013). Optimization of Chemotherapy for Younger Patients with Acute Myeloid Leukemia: Results of the Medical Research Council AML15 Trial. J. Clin. Oncol..

[88] Borthakur G., Kantarjian H., Wang X., Plunkett W.K., Gandhi V.V., Faderl S., Garcia-Manero G., Ravandi F., Pierce S., Estey E.H. (2008). Treatment of core-binding-factor in acute myelogenous leukemia with fludarabine, cytarabine, and granulocyte colony-stimulating factor results in improved event-free survival. Cancer.

[89] Acheampong D.O., Adokoh C.K., Asante D.-B., Asiamah E.A., Barnie P.A., Bonsu D.O., Kyei F. (2018). Immunotherapy for acute myeloid leukemia (AML): A potent alternative therapy. Biomed. Pharmacother..

[90] Cooper S.L., Brown P.A. (2015). Treatment of pediatric acute lymphoblastic leukemia. Pediatr. Clin. N. Am..

[91] Siveen K.S., Uddin S., Mohammad R.M. (2017). Targeting acute myeloid leukemia stem cell signaling by natural products. Mol. Cancer.

[92] Huber B., Eberl L., Feucht W., Polster J. (2003). Influence of Polyphenols on Bacterial Biofilm Formation and Quorum-sensing. Z. Nat. C.

[93] Karakaya S., Koca M., Yılmaz S.V., Yıldırım K., Pınar N.M., Demirci B., Brestic M., Sytar O. (2019). Molecular Docking Studies of Coumarins Isolated from Extracts and Essential Oils of *Zosima absinthifolia* Link as Potential Inhibitors for Alzheimer’s Disease. Molecules.

[94] Siddique A.B., Ebrahim H., Mohyeldin M., Qusa M., Batarseh Y., Fayyad A., Tajmim A., Nazzal S., Kaddoumi A., El Sayed K. (2019). Novel liquid-liquid extraction and self-emulsion methods for simplified isolation of extra-virgin olive oil phenolics with emphasis on (−)-oleocanthal and its oral anti-breast cancer activity. PLoS ONE.

[95] Milani A., Basirnejad M., Shahbazi S., Bolhassani A. (2017). Carotenoids: Biochemistry, pharmacology and treatment. Br. J. Pharmacol..

[96] Williamson E.M. (2001). Synergy and other interactions in phytomedicines. Phytomedicine.

